# “Brucellupus” in a boy: challenging sle diagnosis in childhood

**DOI:** 10.1186/1546-0096-12-S1-P314

**Published:** 2014-09-17

**Authors:** Valeria Paganelli, Edoardo Marrani, Alice Brambilla, Sabrina Becciani, Teresa Giani, Ilaria Pagnini, Gabriele Simonini, Rolando Cimaz

**Affiliations:** 1 Pediatric Rheumatology, Children Hospital of Florence, Florence, Italy; 2 Anna Meyer, Children Hospital of Florence, Florence, Italy

## Introduction

Systemic Lupus Erythematosus (SLE) is the prototype of systemic autoimmune disorders. Several infectious diseases can mimic autoimmune disorders , eg. Mycobacterium tubercolosis), Parvovirus B19), and Leishmania. Brucella infection has been rarely reported in such differential.

## Objectives

To report the case of a boy who presented signs and symptoms suggestive of SLE but who ultimately turned out to be affected by brucellosis.

## Methods

An 11-yr old boy was referred to our hospital for a 2-month history of low grade fevers, malaise, skin rash on face and arms, headaches, weight loss with lack of appetite, arthralgia, recurrent epistaxis and easy bruising. Photosensitivity over the cheeks, trunk and limbs was reported. A previous laboratory work-up had shown mild leukopenia (WBC 2860/mmc), thrombocytopenia (95000/mmc) and elevated CRP levels.

## Results

On admission the patient was ill-appearing: physical examination revealed an erythematous photosensitive rash on the trunk, a butterfly malar rash, oral ulcers and splenomegaly. Fever, previously intermittent, showed a spiking pattern over time. Complete blood counts revealed mild anaemia (Hb: 10.6 g/dl), leukopenia (3440/mmc), and slight prolongation of clotting times. Infectious disease work-up resulted negative for Bartonella henselae, Leishmania, Parvovirus B19; Salmonella typhi and Salmonella paratyphi serology was slightly positive (1:160 and 1:20 respectively), while an initial serology for Brucella spp was negative. Tuberculosis screening resulted negative. A bone marrow aspiration ruled out lymphoproliferative disorders. Autoantibody profile indicated positive antinuclear antibodies at high titre (1:640 on Hep2 cells, nucleolar pattern), while anti ds-DNA, anti-ENA, antiphospholipid antibodies and lupus anticoagulant resulted negative.

The patient fulfilled 5 ACR criteria for the classification of SLE (cutaneous rash, oral ulcers, photosensitivity, haematological disorder, positive antinuclear antibody) and prednisone was administered (40 mg/day) along with Hydroxycloroquine 200 mg/day. The patient showed a rapid clinical improvement on fever and general conditions, with improvement in complete blood count and coagulation markers. He was discharged with the diagnosis of SLE, but few days later blood cultures results came back positive for Brucella spp and Wright serologies came back positive. Steroid therapy and Hydroxycloroquine was then gradually stopped and antibiotic therapy was introduced (Doxycycline 100 mg bid po for six weeks). After six weeks general clinical conditions further improved, blood cultures resulted negative and laboratory findings normalized. Clinical signs and symptoms also slowly improved, and at the last available follow-up (6 months from diagnosis), clinical and laboratory findings were all normal.

## Conclusion

Our case, despite fulfilling the ACR criteria, showed some unusual findings (increased C4 values, an intermittent fever pattern, negative ENA, anti-dsDNA, and aPL) In our case brucellosis, despite lack of history of dairy product consumption, was confirmed by blood culture in two different blood samples. Brucella spp in ections need so be included in the differential diagnosis of SLE.

## Disclosure of interest

None declared

